# Supplementary material for: Optogenetic stimulation of the VTA modulates a frequency-specific gain of thalamocortical inputs in infragranular layers of the auditory cortex
Source: Sci Rep. 2019 Dec 31;9:20385. doi: 10.1038/s41598-019-56926-6 (PMC6938496; doi:10.1038/s41598-019-56926-6)
Supplement: Supplementary file 1 — Supplementary Figures [file 41598_2019_56926_MOESM1_ESM.pdf]

Supplementary information:

Optogenetic stimulation of the VTA modulates a frequency-specific gain of thalamocortical inputs in infragranular layers of the auditory cortex

Authors: Michael G. K. Brunk*[1], Katrina E. Deane[1], Martin Kisse[1], Matthias Deliano[1], Silvia Vieweg[1], Frank W. Ohl[1,2,3], Michael T. Lippert[1,2], Max F. K. Happel*[1,3]

[1] Department of Systems Physiology of Learning, Leibniz Institute for Neurobiology, 39118 Magdeburg, Germany
2 Center for Behavioral Brain Sciences (CBBS), 39106 Magdeburg, Germany
3 Institute for Biology, Otto-von-Guericke-University, 39120 Magdeburg, Germany
* Corresponding authors

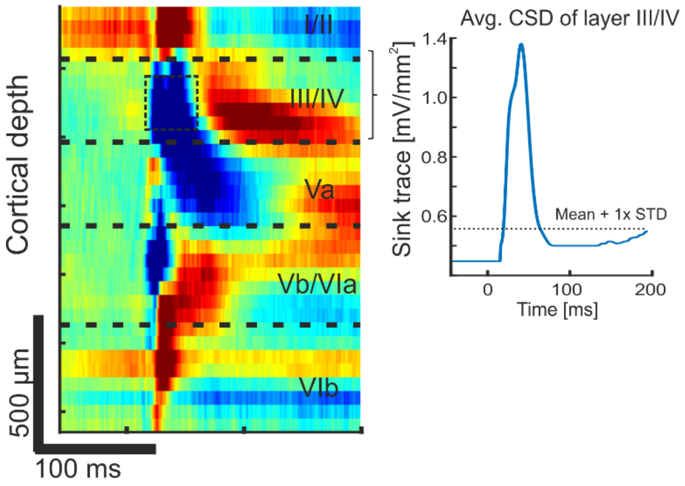

Figure S2- Assignment of layers to CSD channels and semi-automatic detection of sink-activity

Based on previous studies, earliest tone-evoked components are related to layers Vb/VIa and III/IV [18,32]. Subsequent synaptic activity is conveyed to layer Va and VIb, and layers I/II, as indicated in the representative example. Channels assigned to individual layers were averaged and activity attributed to sources (positive values) were dismissed. For visual purposes, a corresponding average-sink trace from layer III/IV was inverted. Based on the pre-stimulus baseline signals (200 ms), the baseline mean value + 1 standard deviation (STD) was calculated as response threshold for each layer-dependent CSD trace. A deflection that surpassed a 1-fold STD was used to determine the onset and offset of early and late sink activations (see Materials and Methods). Significant sink onset activity within the first 50 ms after tone onset was assigned as early activity, while later onsets were assigned as late activity.

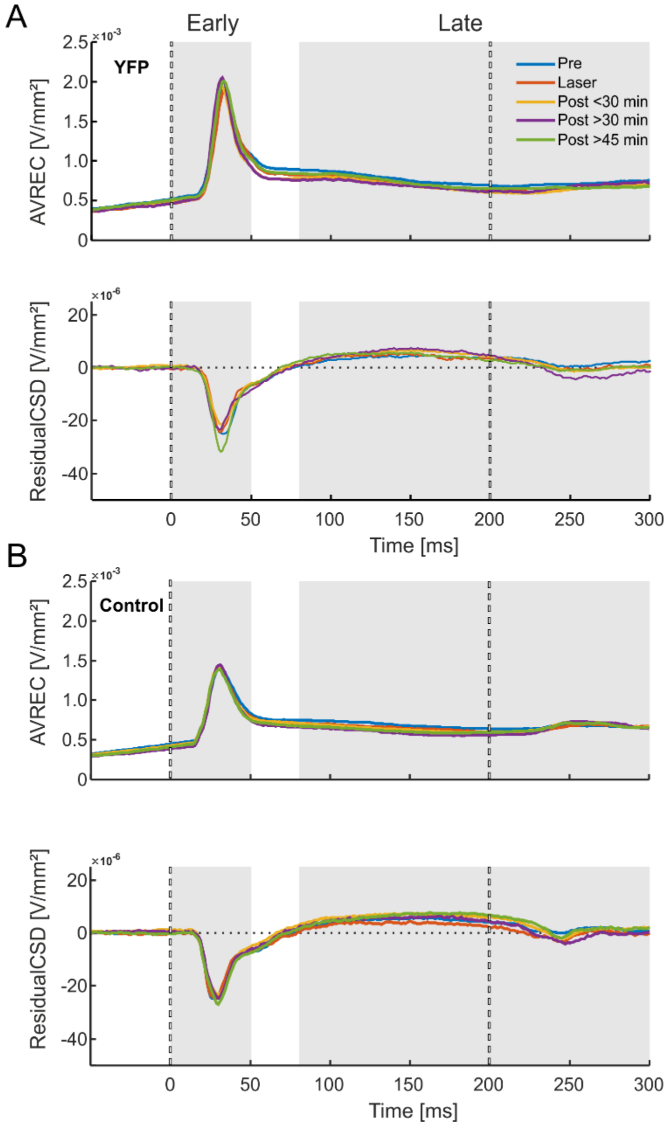

Figure S3 - Grand average AVREC and ResidualCSD traces for YFP and control groups

(A) AVREC and ResidualCSD traces of the YFP group (n=7) for binned pre-measurements, laser-measurement, post-measurements < 30 min, post-measurements > 30 min and post-measurements > 45 min. Dashed lines indicate tonal on- and offset. Areas for early and late RMS calculations are underlined with gray. Group sorting was performed according to the BF of the granular sink peak amplitudes. (B) AVREC and ResidualCSD traces of the control group (n=7) for binned pre-measurements, laser-measurement, post-measurements < 30 min, post-measurements > 30 min and post-measurements > 45 min. Dashed lines indicate tonal on- and offset. Areas for early and late RMS calculations are underlined with gray. Group sorting was performed according to the BF of the granular sink peak amplitudes.

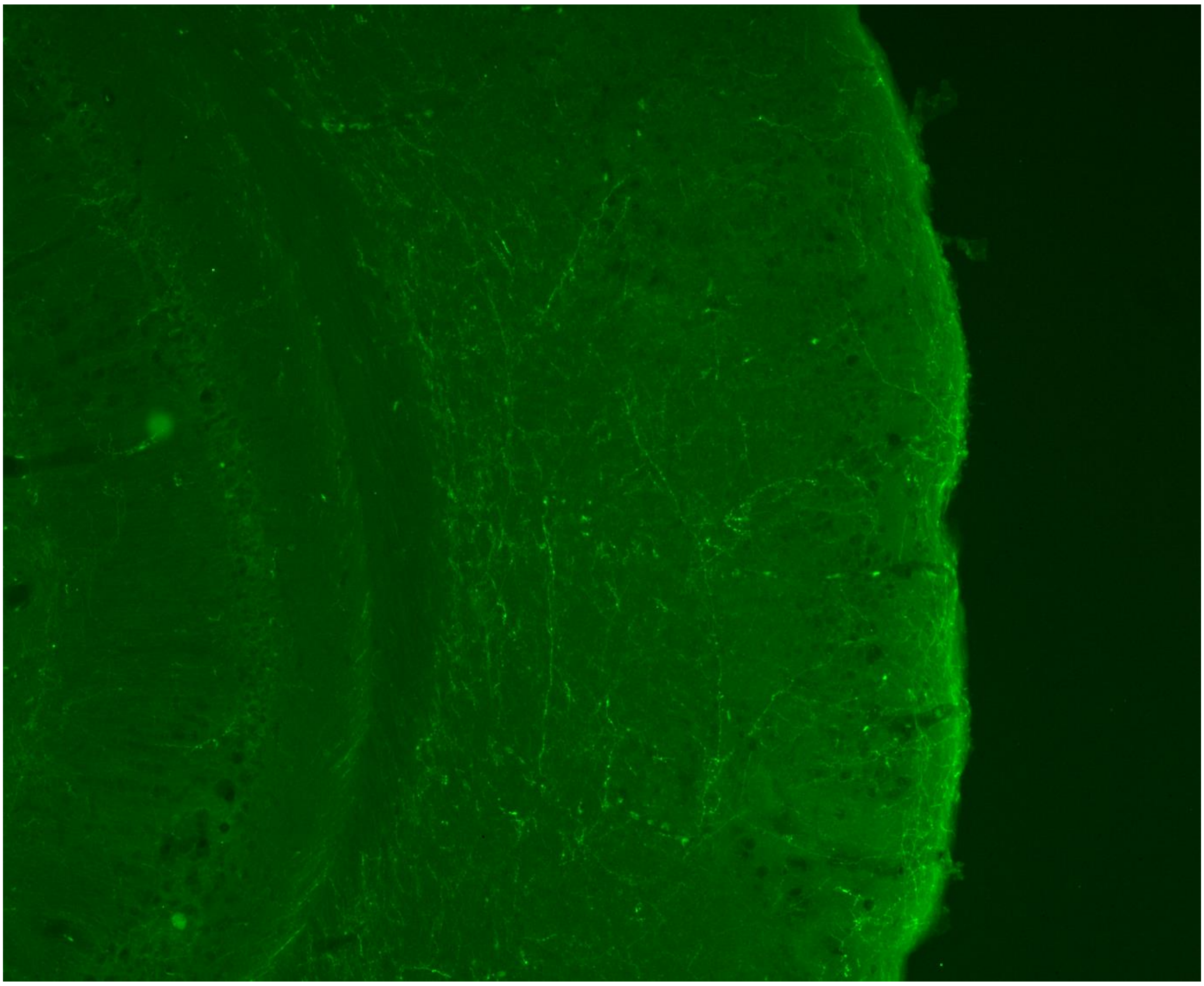

Figure S4 – Corresponding cortical section of Fig 1B

High-resolution image of the ACx-axonal projections of the VTA after transduction with the construct (from Figure 1 B).
